# Factors associated with mental health outcomes in a Muslim community following the Christchurch terrorist attack

**DOI:** 10.1192/bjo.2024.774

**Published:** 2024-11-13

**Authors:** Caroline Bell, Ruqayya Sulaiman-Hill, Sandila Tanveer, Richard Porter, Shaystah Dean, Philip J. Schluter, Ben Beaglehole, Joseph M. Boden

**Affiliations:** Department of Psychological Medicine, University of Otago Christchurch, New Zealand; Te Kaupeka Oranga, Faculty of Health, Te Whare Wānanga o Waitaha, University of Canterbury, Christchurch, New Zealand; and Primary Care Clinical Unit, School of Clinical Medicine, The University of Queensland, Brisbane, Australia

**Keywords:** Trauma, terror attack, Muslim, PTSD, depression

## Abstract

**Background:**

On 15 March 2019, a white supremacist terrorist attacked two mosques in Christchurch, New Zealand. Fifty-one people were killed and another 40 sustained non-fatal gunshot injuries.

**Aims:**

To examine the mental health of the Muslim community, and individual and exposure-related factors associated with mental health outcomes.

**Method:**

This is the baseline analysis of a longitudinal study of adults from the Muslim community interviewed 11–32 months after the shootings. It included a diagnostic interview (MINI), measures of sociodemographic factors, prior mental health, prior traumatic events, exposure in the attacks, discrimination, life stressors, social support and religious coping. Logistic regression models examined associations with mental health outcomes.

**Results:**

The sample comprised 189 participants (mean age 41 (s.d. = 13); 60% female), and included: bereaved, 17% (*n* = 32); injured survivors 12% (*n* = 22); non-injured survivors, 19% (*n* = 36); family members of survivors, 35% (*n* = 67); and community members without the above exposures, 39% (*n* = 74). Overall, 61% had at least one mental disorder since the attacks. Those bereaved (*P* < 0.01, odds ratio 4.28, 95% CI 1.75–10.49) and survivors, whether injured (*P* < 0.001, odds ratio 18.08, 95% CI 4.70–69.60) or not (*P* < 0.01, odds ratio 5.26, 95% CI 1.99–13.89), had greater odds of post-traumatic stress disorder. Those bereaved (*P* < 0.001, odds ratio 5.79, 95% CI 2.49–13.46) or injured (*P* = 0.04, odds ratio 4.43, 95% CI 1.07–18.28) had greater odds of depression.

**Conclusions:**

Despite unique features of this attack on a Muslim population, findings accord with previous studies. They suggest generalisability of psychopathology after terror attacks, and that being bereaved or directly experiencing such events is associated with adverse mental health outcomes.

**Trial registration number:**

The study is registered on the Australian NZ Clinical Trials Registry (ACTRN12620000909921).

On 15 March 2019, a white supremacist gunman opened fire in two mosques within Christchurch, New Zealand (NZ) during Friday *juma'a* (congregational) prayers. The attacks resulted in 51 fatalities with a further 40 people being non-fatally shot and many others sustaining minor injuries. At least 250 survivors were present in the mosques, and the gunman live-streamed the attacks, which led to wide and repeated exposure in the Muslim community. The scale and violence of this act of terrorism was unprecedented in modern NZ and is one of the worst mass shootings in peacetime. It specifically inflicted harm on a minority Muslim population and is one of a series of attacks over recent years which have targeted people at their place of worship.^1^

The intentional nature of terrorist acts is particularly psychologically pernicious with greater risk of adverse mental health outcomes compared with non-intentional disasters.^2,3^ Systematic reviews of mental health outcomes after terrorist attacks consistently report high rates of post-traumatic stress disorder (PTSD) and other mental health disorders,^3–6^ although it is notable that most studies are from high income countries despite terror attacks more frequently occurring outside these regions. To our knowledge, no studies have examined the outcomes of a terrorist attack on a population targeted for their Muslim faith in a non-Muslim majority context.

Factors known to be associated with adverse outcome after exposure to a traumatic event include pre-event characteristics (such as demographic variables, mental health history, previous exposure to trauma); event factors (such as greater proximity and intensity of exposure in the attack, acquaintance with someone deceased, physical injury, perception of life being in danger, panic or dissociative responses at the time); and post-event psychosocial factors (such as having less social support, a change in psychosocial resources, further stressful life events).^4–6^

While many studies following terrorist attacks focus on outcomes for individuals directly involved and their family members, more recently, wider impacts on friends and supports have been recognised.^5^ In small, interconnected communities such as that targeted in the March 15 attacks, many people lost close friends, played significant roles in the response and in addition shared experiences as members of a visible minority group.

This is the first phase of a proposed longitudinal study of an inception cohort of survivors and affected Muslim community members following the terror attack on two mosques in Christchurch, NZ. The aims were to: (a) examine the mental health status of this highly exposed group of Muslims; and (b) investigate the individual and exposure-related factors associated with mental health outcomes.

## Method

### Study design and participants

The study employed a mixed-methods design, composed of a quantitative component (clinician-administered clinical interview and diagnostic assessment, and culturally acceptable self-report measures) and a qualitative sub-study (examining subjective experiences after the attacks). Here, only the quantitative component is reported upon. The study was co-designed with Muslim researchers and involved active community engagement, collaboration with local Muslims (who were employed in research roles) and a Muslim reference group (described in detail in the published protocol).^7^

The study was approved by the NZ Health and Disability Ethics Committee (HDEC Reference 19/NTA/147) and is registered on the Australian NZ Clinical Trials Registry (ACTRN12620000909921). Conduct of the study complied with ethical standards for human experimentation as established by the Helsinki Declaration of 1975, as revised in 2008, and was performed in accordance with the ethical standards from HDEC. The study included only participants who provided written informed consent. Participants were free to withdraw at any time without penalty.

Interviews were conducted between February 2020 and December 2021 (11–32 months after the attacks). When the project started, inclusion criteria included being an adult Muslim (18 years or older) and living in the Christchurch region from one of the following groups: survivor present at or near either mosque during the attacks, close relative of one of the 51 people who died or close relative of someone from the survivor group. Following feedback from the community, from April 2021 eligibility was expanded to include all adult members of the Christchurch Muslim community present in the city when the attacks occurred and at the time of interview. Many of these people were also impacted, through loss of friends or by roles providing support to those more directly exposed, by viewing the live-stream and images of the attacks, and by shared experiences as a visible minority group. Exclusion criteria included living outside Christchurch at the time of recruitment (due to challenges accessing clinical pathways in other locations), and aged under 18 years (because of lack of suitability of measures).

### Recruitment and procedures

As no official list was available of those present in the mosques at the time of the attacks, recruitment relied on community engagement and promotion of the project by passive (flyers, social media posts) and active (social connections, attending community events, word of mouth) methods. Participants could contact the research team directly by phone or social media, or through a dedicated website. Interviews were conducted by a clinician (specialist mental health nurse or clinical psychologist) and a Muslim Research Assistant (RA) who could provide interpreter support if required. Interviews were face to face at a location of the participants' choice or online using Zoom with online questionnaires on Qualtrics (a web-based survey platform; Qualtrics, Provo, UT, USA). Study materials were provided in English, and in translation in Arabic, Bangla, Farsi, Turkish, Somali and Urdu, to allow participants to choose their preferred language (or combination of languages). Retail vouchers of NZ$50 were given to partially compensate for time spent participating. Following the interviews, all cases were discussed with a psychiatrist, and if required, referrals to appropriate organisations were facilitated.

The clinical interview included the Mini-International-Neuropsychiatric-Interview (MINI)^8^ and assessed whether participants had a range of mental health disorders before the attacks, in the period since the attacks and currently i.e. at the time they were interviewed. Assessments of disorders before and since the attacks were made from participants’ descriptions of their symptoms that two mental health clinicians felt were at a level to have constituted a disorder at those times. The inclusion of the clinical interview allowed for a more holistic engagement and understanding of the participants' context and needs and informed recommendations for further support/intervention if required. Covariate factors were assessed using self-report questions (detailed account provided in the protocol).^7^ These included sociodemographic factors, self-report measures of prior exposure to traumatic events before the mosque attacks, perceived discrimination,^9^ life stressors (from a list including, for example, housing, finances, immigration), social support^10^ and scores on a religious coping scale developed for the Muslim faith.^11^ Exposure during or from the mosque attacks was assessed by asking participants to respond to as many items as were relevant, including losing a family member (termed bereaved by attacks), being injured (termed injured survivor), being present during the attacks but not injured (termed non-injured survivor) and being a family member of someone present during the attacks (termed family member of survivor).

### Choice of primary outcomes

The primary outcome measures were the rates of mental health disorders since the attacks obtained from diagnostic data from the MINI^8^ and clinical interview. This captured mental health disorders at any time over this period, not just at the time of interview. The MINI was used because it is one of the most commonly used structured diagnostic clinical interviews internationally including in studies from the Arab world,^12^ and in humanitarian aid and global health settings.

### Statistical analysis

In the first stage of the analysis, diagnoses of panic disorder, agoraphobia, social phobia, generalised anxiety disorder (GAD) and obsessive compulsive disorder (OCD) were collapsed into a single category termed anxiety disorder due to shared characteristics and relatively low rates of the specific disorders. Other disorders identified but with rates <1% were omitted from further analyses. This resulted in three mental health disorders for analysis i.e. anxiety disorder, PTSD and major depressive disorder (MDD) in the period since the attacks. In the next step, a series of Spearman correlations were estimated between these three mental health disorders and covariate factors (sociodemographic factors, prior mental health disorder, prior exposure to traumatic events, exposure from attacks, perceived discrimination, life stressors, social support and religious coping). Age, gender, prior mental health disorder and exposure groups were retained in all analyses. Other predictors that were not significantly (*P* < 0.05) associated with any of the three mental health disorder variables were omitted from further analyses. In the third step of the analyses, three separate logistic regression models were fitted to the data, in order to examine factors associated with the three mental health disorders (anxiety disorder, PTSD, MDD). Variable selection took place through two processes. First, we examined the correlation matrix to determine which variables were significantly (*P* < 0.05) associated with at least one of the mental health disorders. Then, we fitted logistic regression models for each of the three mental health disorders using backward and forward variable substitution (threshold *P*-value for variable removal was originally set at 0.5 and reduced by 0.1 on each successive iteration) to arrive at a stable and parsimonious model. A key feature of these analyses involved controlling for prior anxiety disorder, PTSD and MDD using information from the clinical interview. These variables were used to control for autoregressive effects in the analyses (in which prior disorder may have made a disorder following the attacks more likely). In addition, a further Poisson regression analysis was fitted to the data, with the count measure of number of disorders as the dependent variable, and using the same set of associations. Because participants could have one or more mental health disorders, we chose to fit a common model for all three mental health disorders (as well as the ‘any’ outcome, and the negative binomial model for number of disorders). In all cases, models were adjusted for clustering within families, with robust standard errors estimated. Estimates of the odds ratio (for dichotomous mental health outcomes) and the incidence rate ratio (IRR; for the count measure of the number of disorders) and 95% confidence intervals (CIs) for factors associated with mental health outcomes were obtained via exponentiation. All models were fitted using Stata SE version 17.0 (StataCorp, College Station, TX, USA). Further detail about the statistical modelling and procedure is provided in Supplementary 1 available at https://doi.org/10.1192/bjo.2024.774.

## Analytic code and research material availability

The analytic code and research material that support the findings of this study are available on request from the corresponding author, C.B.

## Transparency declaration

The lead author C.B. affirms that the manuscript is an honest, accurate and transparent account of the study and that no important aspects of the study have been omitted

## Results

**Fig. 1:**
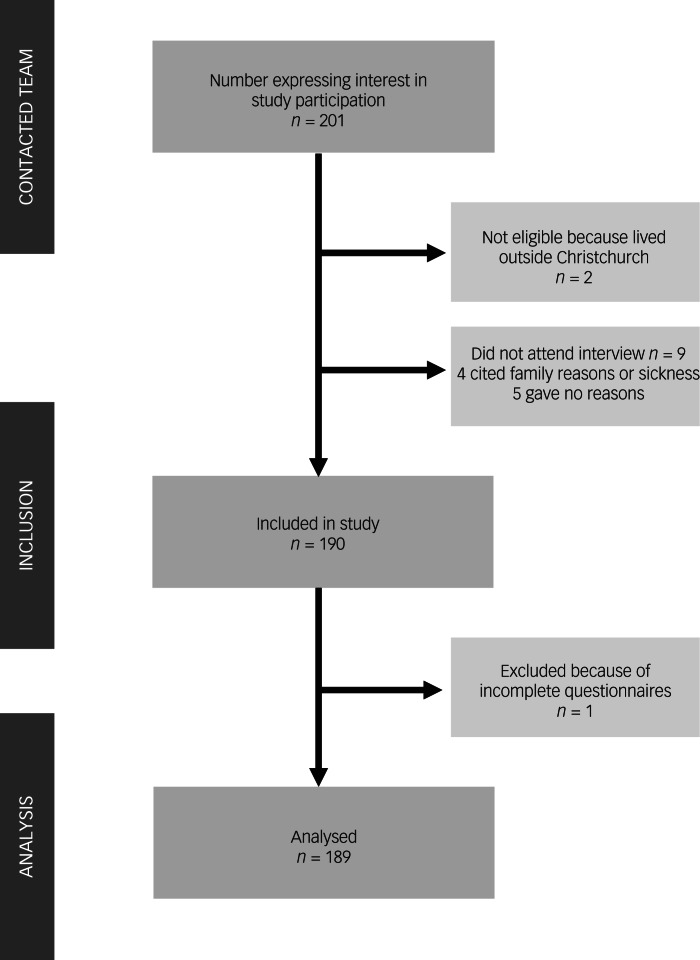
Flow diagram of participant recruitment.

As shown in the participant flow diagram (Fig. 1), the analytic sample comprised 189 individuals. Interviews were conducted between 11 and 32 months (median 25 months, interquartile range (IQR) 11 months) after the attacks. The majority were face to face (80%, *n* = 152), with 40% (*n* = 75) being in participants’ homes. Interviews normally took between 1.5 and 3 h and most were conducted entirely in English (71%, *n* = 135). A total of 26 participants (14%) completed the clinical interview or self-report measures in a combination of their heritage language and English.

### Sociodemographic characteristics

The sample ranged in age from 19 to 74 years (mean 41 years, s.d. = 13), and 60% (*n* = 114) were female. As shown in Table 1, ethnic origin was diverse with the most prevalent ethnicities being Afghanistan, countries from the Middle East, India, Pakistan, Bangladesh and Somalia. The majority of participants (88%, *n* = 169) were not born in NZ, but had lived in NZ for a mean of 14 years (s.d. = 14). Self-reported reasons for coming to NZ included as a migrant (34%, *n* = 64), as a refugee or for family reunification (28%, *n* = 53), as a visitor 6% (*n* = 11) and 22% (*n* = 41) coming for other reasons. Most participants were well educated, with 73% having at least a tertiary qualification (*n* = 138). The majority self-assessed their spoken English and written English as good/very good (75% (*n* = 140) for spoken English and 69% (*n* = 131) for written English).

**Table 1:** Sociodemographic characteristics of sample

Characteristic	% (*n*)
**Gender**	
Male	40 (75)
Female	60 (114)
**Age (years)**	
18–29	20 (42)
30–39	30 (57)
40–49	26 (49)
50–59	13 (25)
60 and above	8 (16)
**Ethnic origin**	
Afghanistan (includes Hazara, Pashtun, Tajik, Turkmenistan)	22 (41)
Middle East (includes Egypt, Algeria, Turkey, Iran)	20 (38)
Indian (includes those of Indian ethnicity from India, Fiji and elsewhere)	13 (25)
African (includes sub-Saharan Africa, e.g. Somalia, South Africa)	11 (21)
Pakistan	8 (15)
Bangladesh	7 (14)
South East Asia (includes Malaysia, Indonesia, Singapore)	7 (14)
Europe (both NZ-born and not) and Others (Māori, Pasifika)	11 (21)
**Self-reported reason for coming to NZ**	
Born in NZ	11 (20)
Migrant	34 (64)
Refugee or for family reunification	28 (53)
Visitor	6 (11)
Other (26 student or spouse of student, 7 for work, with other reasons involving very small numbers i.e. <5)	22 (41)
**Self-reported language proficiency**	
Spoken English	
Very poor	5 (10)
Poor	3 (6)
Average	17 (33)
Good	28 (52)
Very good	47 (88)
Written English	
Very poor	4 (7)
Poor	5 (9)
Average	13 (24)
Good	26 (50)
Very good	43 (81)
**Education**	
No formal qualification (attended school, ESOL class)	13 (24)
Secondary school (e.g. NCEA, IB diploma, overseas school qualification)	14 (27)
Tertiary qualification (e.g. certificate, diploma or trade qualification – less than 3 year course)	15 (29)
Bachelor degree (3–4 year course)	28 (53)
Postgraduate degree (PG diploma, Master's, Doctorate)	30 (56)

NZ, New Zealand; ESOL, English for speakers of other languages; NCEA, National Certificate of Educational Achievement; IB, International Baccalaureate; PG, postgraduate.

### Prior exposure to traumatic events

Exposure to traumatic events before the attacks was common with 80% (*n* = 152) of participants reporting at least one event and 10% (*n* = 19) three or more. The most common was a natural disaster, 60% (*n* = 113), with 44% (*n* = 83) reporting the earthquake series in Christchurch, NZ in 2010/2011 (i.e. about 9 years before the attacks).^13^

### Participant exposure characteristics

**Fig. 2:**
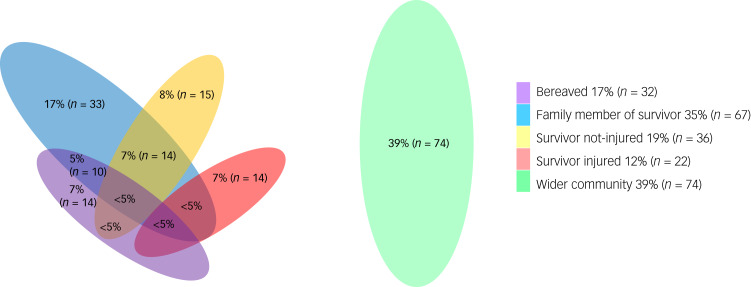
Exposure characteristics: percentage of sample in different exposure categories. Venn diagram is drawn approximately to scale. Numbers of <5% are suppressed to ensure non-identifiability.

The sample included: those who had lost a family member in the attacks, 17% (*n* = 32); injured survivors, 12% (*n* = 22); non-injured survivors, 19% (*n* = 36); family members of a survivor, 35% (*n* = 67); and participants from the wider Christchurch Muslim community, 39% (*n* = 74). As shown in Fig. 2, some participants 23% (*n* = 43) had suffered combinations of these experiences.

### Mental health disorders

Of the 189 participants, 61% (*n* = 115) had at least one mental health disorder (anxiety disorder, MDD or PTSD) at some time following the attacks. In all, 31% (*n* = 58) had an anxiety disorder, 32% (*n* = 61) PTSD and 43% (*n* = 81) MDD. Many had more than one condition; 24% (*n* = 46) had one mental health disorder, 28% (*n* = 53) two and 9% (*n* = 16) all three. Before the attacks, 26% (*n* = 50) of participants had at least one of the mental health disorders, with 2% (*n* = 6) having an anxiety disorder, 4% (*n* = 7) PTSD and 20% (*n* = 37) MDD (Table 2).

**Table 2:** Rates of anxiety disorder, post-traumatic stress disorder (PTSD) and major depressive disorder (MDD) before the attacks, at the time of interview and at some time over the period since the attacks

	Before the attacks % (*n*)	At time of interview % (*n*)	At some time since the attacks % (*n*)
Anxiety disorder	1.6 (6)	27.5 (52)	30.7 (58)
PTSD	3.7 (7)	24.3 (46)	32.3 (61)
MDD	25.4 (37)	27.0 (51)	42.9 (81)
Any mental health disorder (anxiety, PTSD, MDD)	19.6 (72)	47.6 (90)	60.8 (115)
1 disorder	29.6 (56)	22.8 (43)	24.3 (46)
2 disorder	6.9 (13)	18.5 (35)	28.0 (53)
All 3 disorders	1.6 (3)	6.3 (12)	8.5 (16)

Of the 90 participants who had a mental health disorder at the time of interview, 22 were already engaged with treatment. Of the 68 not accessing services, 42 agreed to referral for intervention and support.

### Covariates and specific mental health outcomes

Spearman correlations are shown in Table 3. Having PTSD after the attacks correlated with being bereaved (*r* = 0.17) and being a survivor present during the attacks whether injured (*r* = 0.24) or not (*r* = 0.18). Being a member of the wider community (as opposed to being in one of the other exposure groups) correlated with having a lower rate of PTSD after the attacks (*r* = 0.25). Having MDD after the attacks only correlated with being bereaved by the attacks (0.27).

**Table 3:** Spearman correlations between covariate factors and the three mental health disorders following the attacks (anxiety disorder, post-traumatic stress disorder (PTSD) and major depressive disorder (MDD)

Factors	Anxiety disorder since attacks	PTSD since attacks	MDD since attacks
**Disorder before March 15**			
Anxiety before March 15	0.140	−0.061	0.026
PTSD before March 15	−0.009	0.104	−0.057
MDD before March 15	−0.068	0.002	0.085
Number of disorders	−0.030	0.001	0.055
**Previous trauma exposure**			
Total number of exposures	0.027	0.111	0.088
**Exposure groups**			
Bereaved	−0.056	**0.171**	**0.265**
Survivor injured	0.116	**0.243**	0.086
Survivor not injured	−0.001	**0.184**	0.097
Family member of survivor	−0.061	0.056	0.029
Wider community	0.030	−**0.252**	−0.125
**Demographics**			
Gender	0.047	−0.018	0.047
Age group	−0.133	0.040	0.007
Ethnicity	0.044	0.125	−0.020
Education	0.055	−0.127	−0.039
Coming to New Zealand as refugee	−0.107	0.089	0.005
**Self-reported English proficiency**			
Spoken English	0.001	−0.160	−0.024
Written English	−0.060	−0.158	−0.030
**Measure**			
Religious coping scale	0.016	0.068	−0.076
Perceived discrimination scale	0.09	0.01	−0.01
Social Network Index (social support)	−0.07	−0.07	−0.12
Number of stressors	0.10	−0.04	−0.10

Correlation coefficients shown in bold are statistically significant (*P* < 0.05).

Table 4 shows the percentage of participants with the mental health disorders (anxiety disorder, PTSD, MDD) in each of the categories, for example, 31% (*n* = 57) of participants who did not have PTSD before the attacks were diagnosed as having had PTSD following the attacks.

**Table 4:** Associations with mental health disorders in the period since the attacks

			Mental health disorders in the period since attacks % (*n*)			
		Category (*n*)	Anxiety disorder	PTSD	MDD	No mental health disorder
Gender		Male (75)	28.0 (21)	33.3 (25)	40.0 (30)	42.7 (32)
		Female (114)	31.9 (36)	31.0 (35)	45.1 (51)	36.8 (42)
Age group		18–29 years (42)	40.5 (17)	26.2 (11)	38.1 (16)	45.2 (19)
		30–49 years (106)	30.2 (32)	34.9 (37)	46.2 (49)	39.6 (42)
		50–74 years (41)	22.0 (9)	31.8 (13)	39.0 (16)	43.9 (18)
Prior mental health	Anxiety disorder before attack	No (183)	29.5 (54)	34.7 (60)	42.6 (78)	42.1 (77)
		Yes (6)	66.7 (4)	16.7 (1)	50.0 (3)	33.3 (2)
	PTSD before attack	No (182)	30.8 (56)	31.3 (57)	43.4 (79)	42.3 (77)
		Yes (7)	28.6 (2)	57.1 (4)	28.6 (2)	28.6 (2)
	MDD before attack	No (152)	32.2 (49)	32.2 (49)	40.8 (62)	43.4 (66)
		Yes (37)	24.3 (9)	32.4 (12)	51.4 (19)	35.1 (13)
Number of exposures to traumatic events before attacks		0–1 (119)	29.4 (35)	29.4 (35)	40.3 (48)	43.7 (52)
		2–3 (62)	32.2 (20)	33.9 (21)	45.2 (28)	38.7 (24)
		4–5 (8)	37.5 (3)	62.5 (5)	62.5 (5)	37.5 (3)
Exposure	Survivor injured	No (167)	28.7 (48)	24.9 (47)	41.3 (69)	44.3 (74)
		Yes (22)	45.5 (10)	63.6 (14)	54.5 (12)	22.7 (5)
	Survivor not injured	No (153)	30.7 (47)	28.1 (43)	40.5 (62)	44.4 (63)
		Yes (36)	30.6 (11)	50.0 (18)	52.8 (19)	30.6 (11)
	Family bereaved	No (157)	31.8 (50)	28.7 (45)	36.9 (58)	45.2 (71)
		Yes (32)	25.0 (8)	50.0 (16)	71.9 (23)	25.0 (8)
	Family member of survivor	No (122)	32.8 (40)	18.5 (37)	41.8 (51)	42.6 (52)
		Yes (67)	26.9 (18)	35.8 (24)	44.8 (30)	40.3 (27)
	Wider community	No (115)	29.6 (34)	41.7 (48)	47.8 (55)	36.5 (42)
		Yes (74)	32.4 (24)	17.6 (13)	35.1 (26)	50.0 (37)

PTSD, post-traumatic stress disorder; MDD, major depressive disorder.

In the third step of the analyses, a series of three logistic regression models were fitted to the data, in order to examine the associations with mental health outcomes over the period following the attacks, controlling for prior anxiety disorder, PTSD and MDD. Table 5 shows odds ratio and IRR, 95% CIs and tests of significance for the final fitted models of anxiety disorder, PTSD, MDD and the total number of disorders. Odds ratios are provided for measures of prior disorders and exposure. Those who were female, younger, injured survivors or had a prior history of an anxiety disorder had greater odds of having an anxiety disorder following the attacks. Those who had lost a family member, were survivors of the attacks whether injured or not, or who had greater exposure to traumatic events before the attacks had greater odds of being diagnosed with PTSD. Those who had lost a family member or were survivors injured in the attacks had greater odds of being diagnosed with MDD. Participants who were survivors, whether injured or not, had lost a family member in the attacks, were female or who had greater exposure to traumatic events before the attacks had higher rates of multiple disorders.

**Table 5:** Multivariable models of the associations with mental health outcomes since the attacks

		Anxiety disorder			MDD			PTSD			Total number of disorders		
		OR	95% CI	*P*	OR	95% CI	*P*	OR	95% CI	*P*	IRR	95% CI	*P*
Gender		2.98	1.25–7.11	**0.01**	1.54	0.64–3.72	0.34	1.90	0.76–4.76	0.17	1.49	1.08–2.05	**0**.**02**
Age group		0.61	0.39–0.98	**0**.**04**	0.86	0.51–1.45	0.57	0.94	0.52–1.69	0.83	0.89	0.73–1.08	0.21
Exposure	Family bereaved	0.76	0.28–2.02	0.58	5.79	2.49–13.46	**<0**.**001**	4.28	1.75–10.49	**<0.01**	1.67	1.24–2.23	**<0**.**01**
	Survivor injured	7.14	1.85–27.59	**<0**.**01**	4.43	1.07–18.28	**0**.**04**	18.08	4.70–69.60	**<0**.**001**	3.05	1.87–5.00	**<0**.**001**
	Survivor not injured	2.12	0.80–5.62	0.13	2.40	0.92–6.23	0.07	5.26	1.99–13.89	**<0.01**	1.81	1.29–2.56	**<0**.**01**
	Family member of survivor	0.54	0.17–1.76	0.31	1.12	0.43–2.93	0.62	1.88	0.66–5.32	0.24	1.02	0.69–1.50	0.93
Prior mental health	Anxiety disorder before March 15	10.32	1.74–61.17	**0**.**01**	1.33	0.13–14.08	0.81	0.37	0.52–2.59	0.31	1.37	0.84–2.22	0.19
	MDD before March 15	0.54	0.23–1.29	0.17	1.49	0.53–4.19	0.45	0.56	0.22–1.44	0.23	0.87	0.62–1.21	0.36
	PTSD before March 15	1.48	0.21–10.29	0.69	0.19	0.03–1.21	0.08	2.50	0.58–10.72	0.22	0.96	0.58–1.57	0.70
Prior number of traumatic events		1.12	0.73–1.72	0.61	1.38	0.93–2.04	0.11	1.59	1.03–2.45	**0**.**04**	1.16	1.01–1.33	**0**.**03**

MDD, major depressive disorder; PTSD, post-traumatic stress disorder; OR, odds ratio; IRR, incidence rate ratio.

Items in bold are statistically significantly different (*P* < 0.05).

## Discussion

This study examined rates of mental health disorders in the affected Muslim community 11–32 months after terrorist shootings in two places of worship. Since the attacks, 61% of the 189 participants had at least one mental health disorder. The most common condition was MDD (43%), followed by PTSD (32%) and anxiety disorder (30%). Comorbidity was common, with 28% of the total sample having two disorders and 9% all three. The high rates of mental health disorders found in our study are in the range reported after terrorist attacks involving non-Muslim survivors in Western contexts.^5^ There has been limited comparison of mental health effects of terrorist attacks in different cultures, with one study comparing outcomes after bombing attacks in Oklahoma, USA and Nairobi, Kenya reporting no difference in mental health disorders after the incidents, although those in Nairobi relied more on religious support.^14^ Taken together, these findings suggest generalisability of psychopathology after terrorist attacks across different populations, contexts and cultures. Our study was conducted 11–32 months after the attacks, and although longitudinal studies generally report a trend of reducing rates over time, the findings confirm those from studies after high exposure events over a similar time period. They also highlight that although PTSD is well recognised after exposure to traumatic events, depression and anxiety also occur, and comorbidity is common.^15^

This terror attack resulted in extreme levels of trauma exposure. We report that different exposures had differential mental health sequelae, confirming previous literature.^16,17^ Being an injured survivor was associated with having an anxiety disorder, PTSD, MDD and having a greater number of disorders after the attacks. This accords with previous work showing that suffering a physical injury in a terrorist attack, regardless of severity, is strongly associated with mental health impacts including PTSD,^18^ and highlights the importance of screening for mental health impacts in this group in addition to their physical health needs. We also report that being directly exposed to the attack, even if not injured, was associated with PTSD and having a greater number of disorders. This is likely to relate to the perceived threat to life and being a direct witness to horrific experiences.^19^ While this has been reported previously, it is important to emphasise because the mental health needs of this group of people may not be prioritised in comparison to those bereaved or injured. This can lead to inequity in access to support and entitlements, which can potentially become sources of additional stress.^20^ Our finding that being bereaved by the attacks was associated with PTSD, MDD and having a greater number of disorders also confirms previous literature. While fewer studies examine the mental health impacts of bereavement from a terror attack, there are consistent reports that losing a loved one in such a violent manner is associated with a high risk of developing mental health problems.^21^ We also suggest that the association between bereavement and PTSD supports the expansion of DSM 5 criterion A for PTSD to include ‘learning that the traumatic events occurred to a close family member’. The impacts of terror attacks on those indirectly impacted, such as relatives and friends, has suggested an exposure gradient with different types of exposure having different outcomes,^5^ and our findings confirm this. It has been proposed that different disaster exposures and experiences may selectively contribute to the development of specific psychopathology.^15,22^ Our findings also support this, with MDD more likely to develop after loss of a family member and being injured, and PTSD more likely after the personal experience of physical endangerment and/or injury or loss of a family member.

Our study investigated the role of pre-existing factors on mental health outcomes. As previously reported, we found associations between prior exposure to traumatic events and PTSD^6,23–26^ and a greater number of disorders after the attacks. However, in contrast no association was found between prior exposure and anxiety disorders or MDD.^27^ It is possible that this may be explained by the high rates of prior exposure in our participants (80% having been exposed to at least one traumatic event before the attacks). Contrary to most previous studies,^28–30^ we found that being female was associated with having only an anxiety disorder and a greater number of disorders after the attacks, and not PTSD or MDD.^28–30^ A possible explanation for this may be the sense of collectivism and peer support amongst Muslim women in the context of this attack. The extant literature after trauma in general reports that prior mental health difficulties predict the development of PTSD.^23,25,26^ Before the attacks, 26% of participants in our study had at least one mental health disorder, with MDD being the most prevalent condition. Contrary to previous findings, the only association with prior mental health in our study was having an anxiety disorder after the attacks being associated with a prior history of this disorder. It is also of note that no association was found with other sociodemographic factors such as ethnicity, self-reported assessment of the English language, years in NZ and self-reported reasons for being in NZ (including the 28% of participants from refugee background) and mental health outcome.

We also investigated factors that occur in the aftermath of terror attacks or traumatic events which have been reported to influence outcomes. These include associations between secondary life stressors (recently defined as prior life circumstances and/or societal responses to the disaster/events)^31^ and experiences of perceived discrimination^32^ with adverse mental health outcomes, as well as social support and the use of adaptive coping strategies with more positive outcomes.^2,6,23,27,28^ In contrast to the extant literature, no such associations were found in our study. It is possible that this may be explained by the unique context of the attacks occurring at a place of worship involving individuals from a small and highly interconnected community. Previous studies have suggested that religious engagement in general, and specifically in the context of these attacks, with the Muslim faith, may buffer the negative impacts of stress and discrimination.^33–35^ Contextualising incidents within a broader Islamic framework (part of Allah's plan) or viewing them as trials where they are judged by their response may help facilitate understanding and acceptance, potentially resulting in spiritual growth. Seeking solace in Allah^36^ and the belief that the deceased are martyrs with high status residing in heaven may also provide comfort. In our study, almost all participants had high scores on the religious coping scale suggesting strong engagement with the Muslim faith; however, this limited the examination of correlations with mental health outcomes. Similarly, the interconnected nature of the community was reflected in high scores across the sample on measures of social support, which also limited examination of this factor in relation to mental health outcomes. In addition, government and other agency support for housing and financial and immigration issues could also have potentially mitigated some of the adverse impacts of secondary stressors.

## Strengths and limitations

A strength of the study included its participatory design which included local Muslim engagement and collaboration at every stage, and careful consideration of factors such as logistic barriers and cultural-specific issues. Through this and extensive community networking, we were able to recruit nearly 200 participants.

However, the study has some limitations. The absence of a list of people with different exposures meant that we are unable to precisely quantify the percentage of participants recruited from each exposure category (51 people were killed and estimates are that a further approximately 250 people were present in the two mosques, of whom 40 sustained bullet injuries). Therefore, due to selection bias, the rates of disorder we report may not accurately reflect rates in the affected community. This does not, however, impact the analysis of factors associated with mental health disorders. The study included a relatively small total sample of the Muslim population of Christchurch, although the age distribution, diversity of ethnicities and country of birth were similar to those reported in census data for the region in 2018. The study did not include a non-exposed control group comparison because funding for the project was not sufficient to support the development of a matched control group that did not have significant overlap with those who were more directly affected i.e. from outside Christchurch. It is possible that participants may have under-reported some prior exposure measures, for example sexual abuse because of cultural concerns about social acceptability, although we attempted to mitigate for this by grouping sexual assaults with other types of adversity. The interviews were conducted over a 20-month timeframe as a result of unavoidable delays in data collection because of the court case and sentencing of the gunman, a Royal Commission of Inquiry into the attacks, and the COVID-19 pandemic and associated lockdowns. While this timeframe is quite wide, there was no evidence that the elapsed time between the exposure and interview influenced the analyses, except perhaps in a conservative manner, in that the longer the time which had elapsed since the attacks, the less likely that participants would have either MDD or PTSD.

On the advice of the Muslim reference group and reflecting our trauma-informed approach, we did not ask participants to recount their experiences on the day of the attacks. This meant that we did not include measures previously found to have some association with mental health outcomes such as peritraumatic distress. The focus of this study was on mental health disorder outcome, and we are aware that presentations which are subthreshold for a diagnosis are also common after traumatic events. The rationale for this was based on the study design which allowed for the capture of diagnoses over the period since the attacks, rather than relying on self-report of symptoms at the time of interview. However, this may have meant that associations with subthreshold symptom presentations were not included. Findings reported here are cross-sectional. However, this is the first phase of a longitudinal study with the second phase planned for 5 years after the attacks. The impact of being Muslim on mental health outcomes is recognised as complex.^31^ As discussed, it may have beneficial effects with established associations between religious engagement and positive health outcomes.^32–34^ However, it is important to recognise that there are also potential negative influences, particularly in non-Muslim majority contexts.^31^ These may include stigma about mental health, as well as stress associated with experiences of Islamophobia, marginalisation and challenges with acculturation. Our study did not examine the impact of these factors on mental health outcomes.

## Clinical relevance

Our study involved a participatory approach being co-designed with Muslim researchers and including active engagement with the community at every stage. This was a key factor in the acceptability of the research and for it to be of meaning to participants and the community

Studies consistently report high rates of adverse mental health outcomes after terrorist attacks, although it is notable that most studies to date have been conducted in high income countries.^2–6^ This is the first to examine mental health outcomes after a terrorist attack targeting a Muslim population in a non-Muslim majority context. The findings accord with previous literature with high rates, not only of PTSD (32%), but also other conditions, particularly MDD (43%) and anxiety disorders (31%). They also highlight the association between the type and proximity of exposure and outcome. Suffering a physical injury in a terrorist attack is associated with PTSD,^18^ anxiety and depressive disorders, emphasising the importance of screening for mental health impacts in this group in addition to addressing their physical needs. However, it is also important to note that being directly exposed to the attack, even if not injured, is also associated with PTSD. The mental health needs of this group of people are often not prioritised in comparison to those injured or bereaved, which can lead to inequity in access to supports and entitlements, and further stress.^20^ Being bereaved by a terror attack is associated with both PTSD and MDD,^21^ suggesting that those who lose a loved one in such a sudden and violent manner should also be prioritised for screening and support. Overall, the consistency of outcomes after terrorist attacks, despite differences in contexts and populations targeted, suggests generalisatibility of psychopathology in the wake of such extreme experiences. Although we were limited in any examination of correlation between Muslim religious coping and mental health outcomes, it was vital that the study was culturally informed throughout.

## Supporting information

Bell et al. supplementary material Bell et al. supplementary material

## Data Availability

Data will not be made available due to confidentiality and potential identifiability of sensitive participant information.
