# Supplementary material for: Factors associated with mental health outcomes in a Muslim community following the Christchurch terrorist attack
Source: BJPsych Open. 2024 Nov 13;10(6):e209. doi: 10.1192/bjo.2024.774 (PMC11698172; doi:10.1192/bjo.2024.774)
Supplement: Bell et al. supplementary material [file S2056472424007749sup001.pdf]

Supplement 1

Factors associated with mental health outcomes in a Muslim community following the Christchurch terrorist attack: Analytic approach

In this Supplement, we explain in more detail the statistical modelling and procedure used in the study (Factors associated with mental health outcomes in a Muslim community following the Christchurch terrorist attack) and the considerations that informed this.

As described in the paper the analysis occurred in stages.

Analysis stages

In the first stage of the analysis, diagnoses of Panic Disorder, Agoraphobia, Social Phobia, Generalised Anxiety Disorder (GAD), Obsessive Compulsive Disorder (OCD), generated via the clinical interview, were collapsed into a single category termed Anxiety Disorder due to shared characteristics and relatively low rates of the specific disorders. Other disorders identified, but with rates <1%, were omitted from further analyses. This resulted in three mental health disorders for analysis i.e. Anxiety Disorder, PTSD, Major Depressive Disorder (MDD) in the period since the attacks.

In the next step, a series of Spearman correlations were estimated between these three mental health diagnoses and covariate factors (sociodemographic factors, prior mental health disorder, prior exposure to traumatic events, exposure from attacks, discrimination, life stressors, social support [Social Network Index] and religious coping). Factors that had correlations $r<0{\cdot}1$ with the mental health outcomes were omitted from further analysis.

In the third step of the analyses, three separate logistic regression models were fitted to the data, in order to examine factors associated with the three mental health outcomes (Anxiety Disorder, PTSD, MDD).

Variable selection took place through two processes. First, we examined the correlation matrix to determine which variables were significantly ($p<0{\cdot}05$) associated with at least one of the mental health outcomes. Then, we fitted logistic regression models for each of the three mental health outcomes using backward and forward variable substitution to arrive at a stable and parsimonious model. In the first iteration of the model, covariates with a threshold p value > .5

were removed from the model, and this threshold was reduced by 0.1 for each successive iteration. In all analyses, we controlled for prior Anxiety Disorder, PTSD and MDD using information from the clinical interview. These variables were used to control for autoregressive effects in the analyses (in which prior disorder may have made disorder following the attacks more likely). In addition, a further Poisson regression analysis was fitted to the data, with the count measure of number of disorders as the dependent variable, and using the same set of associations. Because participants could have one or more mental health disorders, we chose to fit a common model for all three mental health outcomes (as well as the "any" outcome, and the negative binomial model for number of disorders). In all cases, models were adjusted for participants clustering within families, with robust standard errors estimated. Estimates of the odds ratio (OR; for dichotomous mental health outcomes) and the incidence rate ratio (IRR; for the count measure of the number of disorders) and 95% confidence intervals (CIs) for factors associated with mental health outcomes were obtained via exponentiation. All models were fitted using Stata SE version 17.0 (StataCorp, College Station, TX, USA).

Discussion of statistical modelling and procedure

In fitting these models, it proved necessary to employ a process of variable selection,[1] on the basis of the following considerations. The first was of course the underlying assumptions and empirical distributions of the dependent measures, which were dichotomous classifications (hence the use of logistic regression). The second consideration was sample size; because there was only a relatively small sample (n = 189), the use of large numbers of variables, or the inclusion of a large number of variables that did not contribute particularly well to explanatory power increased the risk of model pathology (model pathology is indicated by large increases in standard errors of estimate, which in turn leads to increasing imprecision [very wide confidence intervals] in modelling). We therefore adopted a three-stage screening process. This three-stage process initially involved choosing variables that would be included in all models as a matter of course – these included age, gender, and the existence (yes/no) of the mental health disorder prior to, but not during the period of exposure (March 15th terrorist attack), which we refer to as "non-contiguous disorder". Therefore, in modelling major depression, prior non-contiguous major depression was controlled for; for modelling PTSD, prior non-contiguous PTSD was controlled for.

For the remaining variables, we conducted correlations, and dropped those independent variables with negligible associations ($p > \cdot.10$). Then we used backward and forward variable substitution to arrive at a stable and parsimonious model. Due to this process, a subset (and mix) of independent variables were used. Backward and forward variable selection is a commonly-used technique in regression modelling to choose the most relevant variables for inclusion in the final model, and can be found in many publications. Advantages of this approach include [1]:

- Automated process: Stepwise selection automates the process of variable selection by iteratively adding or removing variables based on predefined criteria, reducing the need for manual intervention.
- Efficient use of variables: Stepwise selection aims to find the best subset of variables that contribute most to the model's predictive power, potentially leading to a more parsimonious model with improved interpretability.
- Handles large numbers of predictors: Variable selection processes can handle datasets with a large number of predictors more efficiently than manual selection methods, reducing the computational burden.
- Flexible: Stepwise selection allows for a flexible approach to variable selection by incorporating both forward and backward steps, allowing the model to adapt to different types of data.

However, this approach has recognised disadvantages, which include:

- Risk of overfitting: Stepwise selection can lead to overfitting, especially when using criteria such as p-values or information criteria to determine variable inclusion or exclusion. It may select variables that are statistically significant in the sample but lack predictive power in new data.
- Dependence on criteria: The final model selected by stepwise selection can vary depending on the criteria used for variable selection. Different criteria may lead to different final models, making it challenging to interpret results consistently.

- Does not guarantee the best model: Stepwise selection aims to find a good model rather than the best model. It may miss important interactions or nonlinear relationships among variables that are not captured by the stepwise procedure.

Ultimately, for our research questions and dataset, we considered the advantages of this approach outweighed the disadvantages. However, we also acknowledge that other researchers may have taken a different approach.

References

1. Heinze G, Wallisch C, Dunkler D. Variable selection: a review and recommendations for the practicing statistician. Biometrical Journal 2018;60(3):431-449
